# Five Year Clinical, Radiographic and Soft Tissue Profilometric Outcomes at Two Narrow‐Diameter Implants to Replace Missing Maxillary Lateral Incisors

**DOI:** 10.1111/clr.70010

**Published:** 2025-07-27

**Authors:** Andrea Roccuzzo, Jean‐Claude Imber, Jakob Lempert, Leonardo Mancini, Simon Storgård Jensen

**Affiliations:** ^1^ Shanghai Perio‐Implant Innovation Center, Institute for Integrated Oral, Craniofacial and Sensory Research, Ninth People's Hospital Shanghai Jiao Tong University School of Medicine Shanghai China; ^2^ College of Stomatology, National Center of Stomatology, National Clinical Research Center for Oral Diseases, Shanghai Key Laboratory of Stomatology Shanghai Jiao Tong University Shanghai China; ^3^ Department of Periodontology, School of Dental Medicine University of Bern Bern Switzerland; ^4^ Department of Oral and Maxillofacial Surgery Copenhagen University Hospital Copenhagen Denmark; ^5^ Clinic of Reconstructive Dentistry, Center of Dental Medicine University of Zurich Zurich Switzerland; ^6^ Research Area Oral Surgery, Section for Oral Biology and Immunopathology, Institute of Odontology, Faculty of Health and Medical Sciences University of Copenhagen Denmark

**Keywords:** clinical research, clinical trials, dental implants

## Abstract

**Objectives:**

To compare the 5‐year outcomes in patients with congenitally missing maxillary lateral incisors (MLIs) rehabilitated with two different narrow‐diameter implants (NDIs).

**Materials and Methods:**

One‐hundred patients rehabilitated with a cement‐retained bi‐layered zirconia single‐unit crown on either a Ø2.9 mm (Test) (*n* = 50) or a Ø3.3 mm (Control) (*n* = 50) (T1) were assessed at 1‐, 3‐, and 5‐year follow‐up (T2, T3, T4). Clinical, radiographic, patient‐reported outcome measures (PROMs), biological/technical complications, and esthetic ones were recorded. After the acquisition of intraoral optical scans (IOS) (T4), three different soft tissue profilometric profiles (linear, concave, and convex) were identified.

**Results:**

At T4, 66 patients (*n* = 33 per group; drop‐outs *n* = 33; implant survival rate: 99%; early implant loss *n* = 1) were evaluated. Between T1 and T4, crestal bone level (CBL) changes at Ø3.3 and at Ø2.9 mm implants were comparable (difference: 0.24 mm; *p* > 0.05). Despite the positive recorded esthetic scores (i.e., Score 1–2), at T4, 9.1% of Ø2.9 mm versus 18.2% of Ø3.3 mm implants displayed alveolar process deficiency (Score 3). The frequency of soft tissue profilometric profiles was linear (21.2% vs. 40.6%), concave (72.7% vs. 37.5%) and convex (6.1% vs. 21.9%) (Ø2.9 mm vs. Ø3.3 mm group [*p* > 0.01]). Complications included decementation, ceramic chipping of the incisal edge (3× each), abutment loosening (1×) and a buccal fistula (3×). The statistically significant improved PROMs values at T1 remained stable up to T4 for both groups (*p* > 0.05).

**Conclusion:**

The use of Ø2.9 or Ø3.3 mm implants showed comparable positive long‐term results. Clinicians can rely on both implant types to replace congenitally missing MLIs.

## 1 Introduction

Dental agenesis represents one of the most common developmental anomalies, affecting approximately 7% of the population, with significant negative functional and esthetic implications (Khalaf et al. 2014). Among all teeth, Maxillary Lateral Incisors (MLIs) constitute the second most prevalent tooth group affected by this condition, accounting for 24.3% of cases (Khalaf et al. 2014). Managing young patients with MLI agenesis requires a multidisciplinary approach. Treatment options include orthodontic interventions (either space closure through mesialization of canines or space creation), surgical procedures (autotransplantation or dental implants), and prosthetic solutions such as resin‐bonded or fixed dental prostheses (Beyer et al. 2007; Kafantaris et al. 2020; Kern et al. 2025). In recent years, single‐unit implant‐supported crowns have emerged as a preferred treatment modality for congenitally missing MLI replacement (Galindo‐Moreno et al. 2012). In this context, the introduction of narrow‐diameter implants (NDI) with diameters ≤ 3.3 mm has helped clinicians to overcome anatomical challenges. NDIs are indicated in cases with limited mesio‐distal space and root proximity of the adjacent teeth as well as to reduce the need for horizontal bone augmentation at the implant site by reducing the implant bed osteotomy (Roccuzzo et al. 2021). Historically, NDIs were utilized in posterior sites in cases of narrow ridges (Atieh et al. 2025; de Souza et al. 2018; Zumstein et al. 2023). On this topic, a recent long‐term retrospective study with 10–27 years of follow‐up has documented positive survival and success rates (> 95%) (Xu et al. 2025). However, key risk factors affecting the outcomes were identified, including implant length ≤ 10 mm and the use of single crowns versus splinted restorations. In the esthetic zone, the reliability of Ø2.9 mm NDIs both in the short (Roccuzzo et al. 2022; Walter et al. 2023), as well as in the mid‐term (Roccuzzo et al. 2024), has been documented in terms of implant survival rate, clinical, radiographic, and esthetic outcomes. However, due to differences in case selection (i.e., maxilla vs. mandible), implant placement and loading protocols as well as prosthetic workflow (analogic vs. fully digital) a direct comparison among available studies is challenging.

Hence, in light of the few available long‐term data, the present study aims to document the long‐term outcomes of NDIs (i.e., 2.9 or 3.3 mm) in the replacement of congenitally missing MLIs, focusing on the long‐term function, such as biological and technical complications, and soft tissue profilometric outcomes.

## 2 Materials and Methods

This study reports the 5‐year outcomes of two parallel groups, a prospective, non‐randomized, controlled clinical trial conducted at the Department of Oral & Maxillofacial Surgery, Copenhagen University Hospital, Copenhagen, Denmark. Approval to store and handle the data was provided by the Danish Data Protection Agency (approval number: 2012‐58‐0004; extension number: RH‐2016‐314). The investigation was conducted in accordance with the revised principles of the 2013 Declaration of Helsinki. Informed consent was obtained from each patient before the initiation of the study. Patients' recruitment and treatment began in August 2016 (Roccuzzo et al. 2021). The study was registered on ClinicalTrials.gov prior to the collection and publication of the long‐term data (NCT06500923). Data reporting followed the Strengthening The Reporting of Observational Studies in Epidemiology (STROBE) guidelines.

### 2.1 Patients' Allocation, Surgical and Prosthetic Phase

Between 2016 and 2018, patients with congenitally missing MLIs referred to the Department of Oral & Maxillofacial Surgery, Copenhagen University Hospital, Copenhagen, Denmark, and meeting the inclusion criteria were allocated to the Test Group (2.9 mm [Ø2.9 mm]) (Straumann BLT implant, Roxolid, SLActive, Institut Straumann AG, Basel, Switzerland) whenever the Mesio‐Distal space (MD) of the edentulous gap was between 5.9 and 6.3 mm, while all subjects with a MD between 6.4 and 7.1 mm received a 3.3 mm diameter implant [Ø 3.3 mm] (Straumann BLT implant, Roxolid, SLActive, Institut Straumann AG, Basel, Switzerland) (Control Group). At T0 (i.e., implant placement), the surgical procedure encompassed a free‐hand implant placement and the management of peri‐implant bone defects with standardized protocols: in cases of dehiscences and fenestrations, simultaneous contour Guided Bone Regeneration (GBR) as proposed by Buser et al. (2009) (i.e., autogenous bone chips applied on the exposed implant surface in combination with demineralized bovine bone mineral (DBBM) covered by a double‐layer collagen membrane) (Bio‐Oss + Bio‐Gide, Geistlich Pharma AG, Wolhusen, Switzerland) (Buser et al. 2009). Moreover, all sites displaying facial bone wall thickness < 1.7 mm following implant bed preparation, recorded intraoperatively by means of a dedicated caliper, were treated by means of the same approach except for the application of autogenous bone (Roccuzzo et al. 2021). After 3 months of non‐submerged healing, implants were firstly provisionally restored for 3 months with a screw‐retained PolyMethylMethAcrylate (PMMA) single‐unit crown on temporary abutments (Straumann NC and SC, 1–3 mm gingival height). Subsequently, a cemented feldspatic‐ceramic‐veneered‐zirconia (Ceramill ZI cam) crown on an individualized CAD/CAM titanium abutment (Straumann CARES) was designed and delivered (T1 = baseline). All restorations were adjusted to slight or no occlusion, defined whenever a transparent strip with a 65 μm could be dragged between occluding units with little resistance.

Following prosthetic rehabilitation, all patients were scheduled for standardized follow‐up examinations which took place 1 (T2), 3 (T3) and 5 years (T4) after restoration delivery. Details of the study workflow and of the short‐term (T2) (Roccuzzo et al. 2022) as well as the mid‐term (T3) outcomes have been previously reported in detail (Roccuzzo et al. 2024).

### 2.2 Clinical Follow‐Up Examinations (T2‐T4)

At all clinical follow‐up examinations (i.e., T2, T3, T4), the same experienced dental hygienist (V.N.), not blinded to the provided treatment, recorded implant survival as well as peri‐implant probing depth (in mm; six sites per implant) and the following dichotomous variables (0/1): plaque, suppuration, fistula, pain, and necrosis of the adjacent teeth. Moreover, complications at the abutment level (loosening or fracture) and at the reconstruction level (loss of retention, fracture or chipping of the veneering ceramic) were assessed.

At completion of the T4 clinical examination, a digital impression of the upper and lower arches including occlusion registration was taken with a dedicated IOS (TRIOS 3–3 Shape; Copenhagen, Denmark).

### 2.3 Radiographic, Esthetic, and Peri‐Implant Soft Tissue Profilometric Assessment

Details of the radiographic and esthetic assessment were previously provided (Roccuzzo et al. 2022, 2020). Briefly, non‐standardized and non‐individualized digital intraoral periapical radiographs were taken by means of a paralleling technique at all time points (i.e., T0 to T4). Subsequently, all radiographs were imported into a dedicated software (Zen pro, Carl Zeiss AG) and calibration was performed based on known implant lengths (i.e., 10 or 12 mm). Crestal Bone Level (CBL) was calculated as the linear distance between the implant shoulder and the first bone‐to‐implant contact and considered as bone gain (i.e., positive values) or bone loss (i.e., negative values).

The esthetic assessment was performed on clinical pictures (Roccuzzo et al. 2020) at all clinical examinations (i.e., T1 to T4), scoring all parameters according to the Copenhagen Index Score (CIS) (Dueled et al. 2009) (Hosseini and Gotfredsen 2012) (i.e., 1 = optimal; 4 = not‐sufficient). Two periodontists (A.R.; J‐C.I.) not involved in any part of the active treatment or follow‐up examination performed the radiographic and esthetic assessment independently and in duplicate.

**FIGURE 1:**
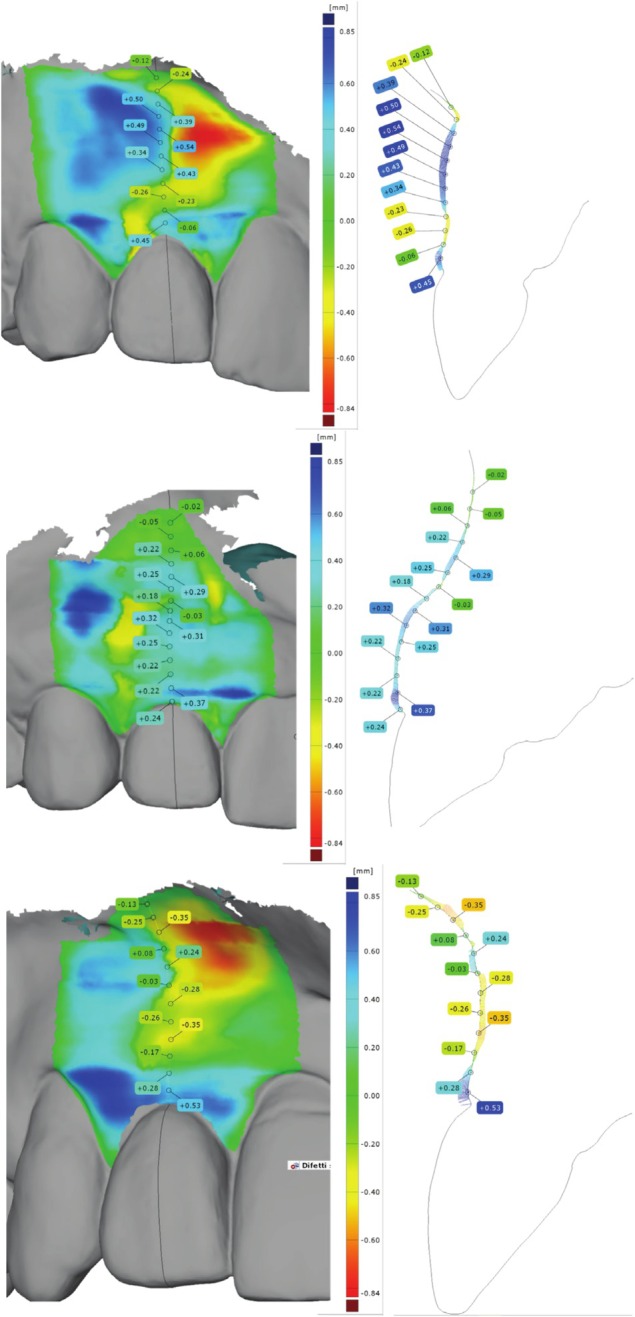
Representative identified peri‐implant profilometric profiles: convex (a), linear (b) and concave (c).

Finally, the STL files recorded by means of intra‐oral scans at T4 were imported and analyzed in a dedicated software (GOM Inspect; GOM, Germany). The 3D surface defect map analysis was performed by an experienced operator (L.M.) according to a previously described and validated approach (Mancini et al. 2024; Mancini, Galarraga‐Vinueza, et al. 2023). Briefly, to establish the assessment framework, a reference plane was defined along the midfacial aspect of the implant site. Two additional planes were then created at the midfacial aspect of the adjacent teeth, ensuring parallel alignment with the initial reference plane. Subsequently, a horizontal cutting plane was introduced perpendicular to the vertical planes of the adjacent teeth, determining the cutting section direction. An automated function within the image analysis software generated a 3D surface defect map, enabling the visualization and quantification of deviations within these planes. These deviations were measured relative to the horizontal cutting plane, indicating whether the soft tissues were positioned more buccally or palatally in the assessed region. To extract quantitative data from the 3D surface defect map, 10 linear divergent points (LDP), at intervals of 0.5 mm each, were identified starting at the peri‐implant soft tissue margin. Positive LDP values indicated that the soft tissues were positioned more buccally (i.e., convex profile) relative to the adjacent dentition profile, while negative LDP values signified a more lingual/palatal (i.e., concave profile) position. Values close to zero showed a linear profile. Finally, sites were categorized based on their 3D surface defect map patterns and LDP measurements (Figure 1).

The impact on oral health‐related quality of life was evaluated using a validated Danish version of the OHIP‐49 questionnaire before prosthetic treatment (i.e., before impression for the temporary crown), at T2 and T4. Patients scored each question with a Likert response scale from 0 (never experienced problem) to 4 (problem experienced very often). The summary of questions 3, 4, 20, 22, 31, and 38 was used to describe the domain “esthetic outcome” (Dueled et al. 2009), while “masticatory function” was expressed by the summary scores of questions 1, 28, 29, and 32 (Goshima et al. 2010).

## 3 Statistical Analysis

In cases with bilateral congenitally missing MLIs, where implants with identical diameters were placed, only one implant was randomly selected for the statistical analysis (www.randomization.com).

### 3.1 Sample Size Calculation

Study primary outcome measure was implant survival rate. Sample size calculation was performed on the secondary outcome parameter peri‐implant CBL reduction according to Hosseini et al. (2013). More specifically, to detect a difference of 0.4 mm and a standard deviation of 0.6, 49 patients per group were needed with alpha (Type I error) = 0.05 and power = 0.9. Using an independent sample *t*‐test, a group size of *N* = 49 was calculated. Patients number was rounded to 50 per group.

### 3.2 Data Analysis

Each patient contributed with one dental implant only and was, therefore, considered as the statistical unit. From the original cohort, only subjects participating in all follow‐up visits (i.e., T1–T4) were included in the present analysis. Descriptive analysis was performed providing absolute and relative frequencies for categorical variables and mean, standard deviation for continuous variables. Normal distribution of the quantitative measures was checked by Kolmogorov–Smirnov test. Mixed ANOVA for repeated measures was conducted to analyze Marginal Bone Level over follow‐up between groups. Corrections based on Greenhouse–Geisser were obtained due to lack of sphericity (Mauchly's test). Bonferroni's post hoc tests were considered for multiple comparisons. The calculated inter‐examiner agreement with Dahlberg's *d* test was in the range of 0.07–0.08 mm and the Intra‐Class Correlation Coefficient (ICC) was estimated at 0.987 providing a very high level of reproducibility of the performed measurements. Similar methodology was considered for OHIP outcomes. Mean Bone Loss between specific timepoints was compared using 2‐sample *t*‐test. Regarding esthetic outcomes, non‐parametric Brunner–Langer's models for longitudinal data were conducted including effects for group, time and interaction. A statistic ATS (Anova‐type) was obtained and pairwise comparisons were based on Mann–Whitney's test (inter‐groups) and Wilcoxon's test (intra‐groups) with Bonferroni's correction. The soft tissue profile was correlated with intra‐operative parameters (i.e., Width of the Alveolar Ridge [WAR], Thickness of the Facial Bone [TFB] after osteotomy and need of GBR) using simple and multiple ordinal logistic regression testing the hypothesis of proportional odds. The relationship between the soft tissue profile and the esthetic scores was analyzed using Kendall's tau‐b test. All tests were two‐tailed, and the level of significance was set at 5%. The statistical analysis was performed by a professional biostatistician not involved in the study with a commercially available dedicated software (SPSS 15.0, Chicago, IL., USA).

## 4 Results

### 4.1 Patients Sample

From the 100 patients originally included and treated within this study, 33 patients per group (dropouts: Ø2.9 mm [*n* = 17] and Ø3.3 mm [*n* = 16]; [*p* = 0.887]) reached T4. At T0, the two groups were comparable with respect to patients' characteristics (i.e., age and gender) and implant length (*p* > 0.05) (data previously reported—Roccuzzo et al. 2022). Reasons for dropout were either unwillingness to attend the follow‐up examination or that the patients had moved abroad. Details of the patients' sample through the study period are provided in Table 1.

**TABLE 1:** Patient count within the two groups (2.9 diameter; 3.3 diameter) throughout the study period.

	2.9 Ø	3.3 Ø	*p*
Baseline (i.e., definitive crown delivery) (T1)	50	49	1.000
1‐year (T2)	47	45	0.715
3‐year (T3)	39	35	0.452
5‐year (T4)	33	33	1.000
Implant loss	0	1	1.000
Drop‐outs	17	16	0.887

*Note:* Chi‐square and Fisher's exact test.

### 4.2 Main Findings (Implant Survival Rate, Crestal Bone Level Changes, Complications)

**FIGURE 2:**
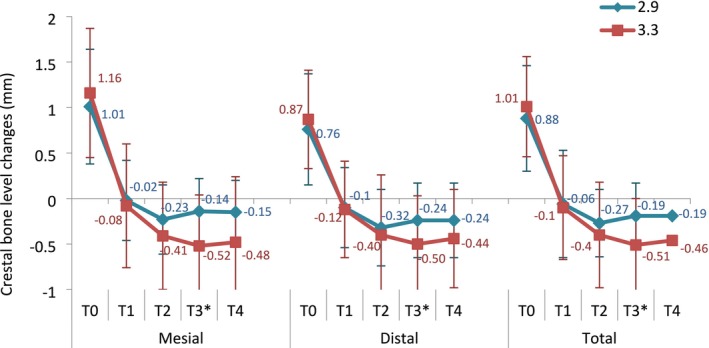
Mean ± SD mesial, distal and total crestal bone level changes over time within the two groups. T0: Implant placement; T1: Final crown delivery (baseline); T2: 1‐year follow‐up; T3: 3‐year follow‐up; T4: 5‐year follow‐up. SD: Standard deviation. *Statistically significant difference: *p* < 0.05. *p*‐values from Bonferroni's test for multiple comparisons of mixed repeated measures ANOVA.

**FIGURE 3:**
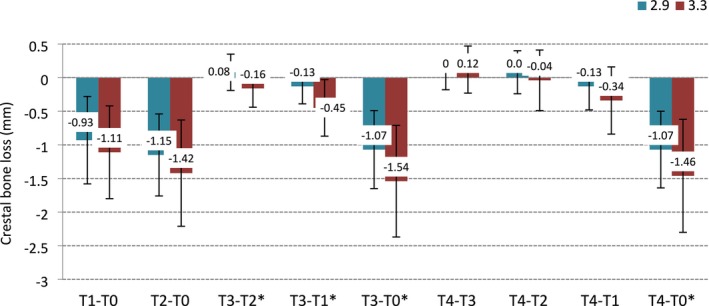
Mean ± SD differences in crestal bone loss between time points within and between the two groups. T0: Implant placement; T1: Final crown delivery (baseline); T2: 1‐year follow‐up; T3: 3‐year follow‐up; T4: 5‐year follow‐up. SD: Standard deviation. *Statistically significant difference: *p* < 0.05. *p*‐values from two‐sample *t*‐test for each outcome (difference between time‐points).

Besides one event of early implant failure within the Ø3.3 mm group, no additional implants were lost (survival rate: 99%; *p* = 1.000; 95% CI: 94.6%–99.9%). With respect to CBL changes, a statistically significant difference between the two groups was detected at T3 (−0.19 mm vs. −0.51 mm Ø2.9 vs. Ø3.3 mm group; *p* = 0.007). However, when analyzing the CBL changes between groups, the only statistically significant difference detected at the latest follow‐up was T4–T0, revealing higher CBL in the Ø3.3 mm than in the Ø2.9 mm (*p* = 0.032). At T4, none of the analyzed implants showed mean CBL > 2 mm. Details of the CBL changes over time as well as the recorded marginal CBL between different time points are represented in Figures 2 and 3.

At T4, mean peri‐implant probing depth values were 2.25 ± 0.29 mm in the Ø2.9 mm group and 2.35 ± 0.36 mm in the Ø3.3 mm group respectively, without any statistically significant difference between groups (*p* = 0.206). Similar results were detected between groups at all previous follow‐ups (T2 & T3) (range 2.48–2.61 mm; *p* > 0.05). Presence of visual plaque was detected around 3 and 4 Ø2.9/3.3 mm implants respectively (*p* > 0.05).

With respect to the recorded technical complications (T1–T4), they accounted for loss of retention (*n* = 3), ceramic chipping of the incisal edge (*n* = 3), and abutment loosening (*n* = 1). Despite the detection of a buccal fistula (*n* = 3), the overall collected clinical and radiographic outcomes were never compatible with a diagnosis of peri‐implantitis.

**TABLE 2:** Frequency of esthetic scores within the Ø2.9 and Ø3.3 mm groups at baseline (T1), 1‐year (T2), 3‐year (T3) and 5‐year follow‐up examination (T4).

		T1		*p*	T2		*p*	T3		*p*	T4		*p*
		Ø2.9 mm	Ø3.3 mm		Ø2.9 mm	Ø3.3 mm		Ø2.9 mm	Ø3.3 mm		Ø2.9 mm	Ø3.3 mm	
		*n* = 33	*n* = 33		*n* = 33	*n* = 33		*n* = 33	*n* = 33		*n* = 33	*n* = 33	
*Symmetry/harmony*	1 2 3	42.4% 36.4% 21.2%	39.4% 54.5% 6.1%	1.000	42.4% 36.4% 21.2%	39.4% 54.5% 6.1%	1.000	40.6% 43.8% 15.6%	34.4% 59.4% 6.3%	1.000	39.4% 45.5% 15.2%	33.3% 60.6% 6.1%	1.000
*Soft tissue color*	1 2 3	48.5% 42.4% 9.1%	45.5% 54.5% 0.0%	1.000	42.4% 42.4% 15.2%	36.4% 60.6% 3.0%	1.000	28.1% 56.3% 15.6%	9.4% 78.1% 12.5%	1.000	30.3% 54.5% 15.2%	15.2% 66.7% 18.2%	0.924
*Papilla index (mes)*	1 2 3	60.6% 39.4% 0.0%	62.5% 37.5% 0.0%	1.000	75.8% 24.2% 0.0%	65.6% 34.4% 0.0%	1.000	59.4% 40.6% 0.0%	56.3% 37.5% 6.3%	1.000	63.6% 27.3% 9.1%	45.5% 39.4% 15.2%	0.574
*Papilla index (dis)*	1 2	84.4% 15.6%	100% 0.0%	0.092	96.9% 3.1%	100% 0.0%	1.000	81.3% 18.7%	87.5% 12.5%	1.000	75.8% 24.2%	72.7% 27.3%	1.000
*Level of the margin*	1 2 3	69.7% 30.3% 0.0%	90.9% 9.1% 0.0%	0.126	69.7% 30.3% 0.0%	87.9% 12.1% 0.0%	0.292	62.5% 37.5% 0.0%	87.5% 12.5% 0.0%	0.088	57.6% 42.4% 0.0%	72.7% 27.3% 0.0%	0.800
*Soft tissue texture*	1 2 3	63.6% 36.4% 0.0%	87.9% 12.1% 0.0%	0.090	84.8% 15.2% 0.0%	93.9% 6.1% 0.0%	0.936	78.1% 21.9% 0.0%	65.6% 28.1% 6.3%	0.894	72.7% 27.3% 0.0%	60.6% 27.3% 12.1%	0.770
*Soft tissue curvature*	1 2	78.8% 21.2%	93.9% 6.1%	0.300	93.9% 6.1%	93.9% 6.1%	1.000	81.3% 18.8%	90.6% 9.4%	1.000	84.8% 15.2%	87.9% 12.1%	1.000
*Alveolar process deficiency*	1 2 3	72.7% 27.3% 0.0%	60.5% 39.4% 0.0%	1.000	66.7% 33.3% 0.0%	54.5% 36.4% 9.1%	0.869	56.3% 40.6% 3.1%	40.6% 37.5% 21.9%	0.309	51.5% 39.4% 9.1%	33.3% 48.5% 18.2%	0.440

*Note:* Inter‐group comparisons: *p*‐values obtained from multiple comparisons between‐groups (2.9 vs. 3.3 mm) for each timepoint using Mann–Whitney's test corrected by Bonferroni. Symmetry/harmony: assessment according to facial midline, tooth axis, contralateral tooth and smile line. Score 1: excellent; score 2: suboptimal but satisfactory; score 3: moderate; score 4: poor symmetry and harmony. Soft tissue score: Score 1: no discoloration, score 2: light greyish discoloration, score 3: distinguishable greyish discoloration, score 4: metal or abutment visible. Papilla index: Score1: papilla filling the entire proximal space; score 2: papilla filling at least half of the entire proximal space; score 3: papilla filling less than half of the proximal space, score 4: no papilla. Level of the margin: assessment of the apically or incisally position of the buccal marginal peri‐implant mucosa in the middle of the implant crown compared to the contralateral tooth or the neighboring teeth. Score 1: match; score 2: slight mismatch; score 3: moderate mismatch; score 4: mismatch. Soft tissue texture: assessment related to the smoother or rougher surface texture of the buccal peri‐implant mucosa compared to natural gingiva at the contralateral tooth or the neighboring teeth. Score1: match; score 2: slight mismatch; score 3: moderate mismatch; score 4: distinct mismatch. Soft tissue curvature: assessment according to the over‐contoured or under‐ contoured buccal marginal peri‐implant mucosa compared to natural gingiva at the contralateral tooth or the neighboring teeth. Score1: match; score 2: slight mismatch; score 3: moderate mismatch; score 4: distinct mismatch. Alveolar process deficiency: assessment related to the concavity or convexity of the buccal peri‐implant mucosa compared to the natural contour of the buccal gingiva at the contralateral tooth or the neighboring teeth. Score1: match; score 2: slight mismatch; score 3: moderate mismatch; score 4: distinct mismatch.

### 4.3 Additional Findings (Esthetic, Profilometric and Patient‐Reported Outcomes)

At none of the follow‐up examinations, statistically significant differences between the Ø2.9 and Ø3.3 mm groups were observed for all evaluated parameters (*p* > 0.05). More in detail, when analyzing frequency changes of esthetic scores over time within each group, both Ø2.9 and Ø3.3 mm groups showed statistically significant impairment in soft tissue color (*p* = 0.014 and *p* = 0.002 respectively) and alveolar process deficiency (*p* = 0.012 and *p* = 0.002 respectively) between T1 and T4. A similar trend was noticed when focusing on the differences between T2 and T4: more specifically, Ø3.3 mm implants demonstrated statistically significant changes in soft tissue color (*p* = 0.04), mesial and distal papilla indices (*p* = 0.015 & *p* = 0.014), soft tissue texture (*p* = 0.007), and alveolar process deficiency (*p* = 0.023), while Ø2.9 mm implants only showed significance in alveolar process deficiency (*p* = 0.034). No statistically significant changes were detected in either group between T3 and T4 (*p* > 0.05) (Tables 2, 3 and 4).

**TABLE 3:** Changes at frequency of esthetic scores within the Ø2.9 and Ø3.3 mm groups (T4‐T1, T4‐T2 and T4‐T3).

	T4‐T1		T4‐T2		T4‐T3	
	Ø2.9 mm	Ø3.3 mm	Ø2.9 mm	Ø3.3 mm	Ø2.9 mm	Ø3.3 mm
	*p*	*p*	*p*	*p*	*p*	*p*
*Symmetry/harmony*	1.000	0.472	1.000	0.472	1.000	1.000
*Soft tissue color*	**0.014**	**0.002**	0.307	**0.004**	1.000	1.000
*Papilla index (mesial)*	1.000	0.055	0.264	**0.015**	1.000	0.173
*Papilla index (distal)*	1.000	**0.014**	0.059	**0.014**	1.000	0.176
*Level of the margin*	0.618	**0.043**	0.307	0.076	0.472	0.076
*Soft tissue texture*	1.000	**0.015**	0.137	**0.007**	0.952	0.307
*Soft tissue curvature*	1.000	0.472	0.539	0.472	0.952	0.952
*Alveolar process deficiency*	**0.012**	**0.002**	**0.034**	**0.023**	0.137	1.000

*Note:* Intra‐group comparisons: *p*‐values obtained from multiple comparisons within‐groups (T4‐T1, T4‐T2, T4‐T3) for each group using Wilcoxon's test corrected by Bonferroni. Bold values indicate statistically significant difference.

**TABLE 4:** Changes of frequency of esthetic scores within the Ø2.9 and Ø3.3 mm groups.

	Time	Group	Time‐Group
*Symmetry/harmony*	0.501	0.790	0.235
*Crown morphology*	0.323	**< 0.001**	0.312
*Crown color*	0.191	0.133	0.772
*Soft tissue color*	**< 0.001**	0.582	0.088
*Papilla index (mesial)*	**0.008**	0.402	0.298
*Papilla index (distal)*	**< 0.001**	0.240	0.342
*Level of the margin*	**0.007**	**0.028**	0.623
*Soft tissue texture*	**0.001**	0.896	**0.004**
*Soft tissue curvature*	0.123	0.292	0.199
*Alveolar process deficiency*	**< 0.001**	0.107	0.514
*Cement excess (0/1)*	**0.042**	0.736	0.306

*Note: p*‐Values obtained from ATS (ANOVA‐type statistic) of Brunner‐Langer's non‐parametric longitudinal models for main effects and interaction. Bold values indicate statistically significant difference.

The frequency of soft tissue profilometric profiles described at T4 were as follows: linear (21.2% vs. 40.6%), concave (72.7% vs. 37.5%) and convex (6.1% vs. 21.9%) within Ø2.9 and Ø3.3 mm groups, respectively (*p* = 0.004). Moreover, the profiles analysis with respect to the analyzed esthetic variable detected a statistically significant association between soft tissue texture scores only within controls (*p* < 0.001), with convex profiles associated with worse soft tissue texture scores. No additional statistically significant associations were observed between profilometric profiles and esthetic parameters in either group (*p* > 0.05) (Table 5). The multiple logistic regression analysis detected only implant diameter as the variable statistically significant affecting soft tissue profile at T4 (OR 0.25; *p* = 0.015; 95% CI: 0.08–0.76) (for details on the simple and multiple logistic regression analysis refer to Tables S1 and S2).

**TABLE 5:** Associations between peri‐implant soft tissue profilometric profiles and esthetic scores at time T4.

		T4			
		Ø2.9 mm (*n* = 33)	*p*	Ø3.3 mm (*n* = 32)	*p*
		convex/linear/concave		convex/linear/concave	
		*n* = 2/*n* = 7/*n* = 24		*n* = 7/*n* = 13/*n* = 12	
*Symmetry/harmony*	1 2 3	0.0/15.4/84.6% 6.7/33.3/60.0% 20.0/0.0/80.0%	0.306	0.0/50.0/50.0% 35.0/35.0/30.0% 0.0/50.0/50.0%	0.147
*Soft tissue color*	1 2 3	0.0/40.0/60.0% 11.1/5.6/83.3% 0.0/40.0/60.0%	0.712	40.0/40.0/20.0% 19.0/33.3/47.6% 16.7/66.7/16.7%	0.825
*Papilla index (mesial)*	1 2 3	4.8/23.8/71.4% 11.1/22.2/66.7% 0.0/0.0/100.0%	0.702	7.1/50.0/42.9% 30.8/30.8/38.5% 40.0/40.0/20.0%	0.132
*Papilla index (distal)*	1 2	8.0/24.0/68.0% 0.0/12.5/87.5%	0.174	13.0/47.8/39.1% 44.4/22.2/33.3%	0.303
*Level of the margin*	1 2	5.3/10.5/84.2% 7.1/35.7/57.1%	0.103	30.4/30.4/39.1% 0.0/66.7/33.3%	0.431
*Soft tissue texture*	1 2 3	8.3/16.7/75.0% 0.0/33.3/66.7% —	0.751	10.5/31.6/57.9% 55.6/33.3/11.1% 0.0/100.0/0.0%	**< 0.001**
*Soft tissue curvature*	1 2	3.6/21.4/75.0% 20.0/20.0/60.0%	0.474	17.9/42.9/39.3% 50.0/25.0/25.0%	0.343
*Alveolar process deficiency*	1 2 3	5.9/23.5/70.6% 0.0/15.4/84.6% 33.3/33.3/33.3%	0.829	30.0/60.0/10.0% 6.3/25.0/68.8% 50.0/50.0/0.0%	0.858

*Note: p*‐Values obtained from Kendall's Tau‐b for comparisons within each group. Bold values indicate statistically significant difference.

Patients reported an increased satisfaction (decreased values) with respect to both esthetic and masticatory domain outcomes between pre‐treatment and T2 without a statistically significant difference between the two groups (*p* > 0.05). More in detail, the mean score for esthetic outcome decreased from 10.4 ± 6.00 to 2.06 ± 3.66 in the Ø2.9 mm group and from 9.24 ± 6.38 to 1.61 ± 2.84 in the Ø3.3 mm group at T4. Similarly, masticatory function scores improved from 3.02 ± 2.76 to 0.44 ± 0.84 in the Ø2.9 mm group and from 4.10 ± 3.04 to 0.58 ± 1.25 in the Ø3.3 mm group. No statistically significant differences were observed between the groups at any timepoint for either esthetic or masticatory domain (*p* > 0.05) (Table 6).

**TABLE 6:** Patient‐reported outcome means of sum scores of questions related to esthetic outcome, masticatory function of prosthetic treatment, before prosthetic treatment, at 1 (T2) and 5‐year (T4) follow‐up.

	Before prosthetic treatment		*p*	T2		*p*
	Ø2.9 mm	Ø3.3 mm		Ø2.9 mm	Ø3.3 mm	
Esthetic outcome^a^	9.93 ± 6.03	9.59 ± 6.41	0.902	3.72 ± 5.97	2.09 ± 4.21	0.294
Masticatory function^b^	2.47 ± 2.39	4.41 ± 3.15	**0.009**	1.13 ± 2.06	0.91 ± 1.61	0.688

	T4		*p*
	Ø2.9 mm	Ø3.3 mm	
Esthetic outcome^a^	2.19 ± 3.74	1.61 ± 2.84	0.716
Masticatory function^b^	0.44 ± 0.84	0.58 ± 1.25	0.518

*Note:* Bonferroni‘s test was used for multiple comparisons from mixed repeated measures ANOVA.

^a^ Esthetic outcome: summary scores of OHIP questions 3, 4, 20, 22, 31, 38.

^b^ Functional outcome: summary scores of OHIP questions 1, 28, 29, 32.

## 5 Discussion

This present clinical study compared the 5‐year outcomes of two NDIs (Ø2.9 mm vs. Ø3.3 mm) for replacing congenitally missing MLIs. Based on the obtained results, it can be stated that both NDIs represent a reliable treatment modality in such clinical scenarios up to 5 years.

Historically, implant survival rate and CBL changes have been the main focus of clinical studies: the 5‐year overall implant survival rate was 99%, with only one early implant failure in the Ø3.3 mm group, which is comparable to those previously reported after at least 5 years of function (Branzen et al. 2015; Galindo‐Moreno et al. 2017; Scarano et al. 2019). When focusing on CBL changes, limited and comparable CBL values between groups at the 5‐year follow‐up were observed, with the Ø3.3 mm group demonstrating slightly greater bone loss (−0.34 mm) compared to the Ø2.9 mm group (−0.10 mm) (difference: 0.24 mm; *p* > 0.05). One possible reason for that might be ascribed to the less extensive osteotomy for Ø2.9 mm implants resulting in reduced surgical trauma and greater blood supply maintenance (Monje, Roccuzzo, et al. 2023). However, from a clinical perspective, it must be recalled that a radiographic evidence of peri‐implant CBL within 0.5 mm has to be considered as direct evidence of implant success (Ribeiro Dos Reis et al. 2025).

**FIGURE 4:**
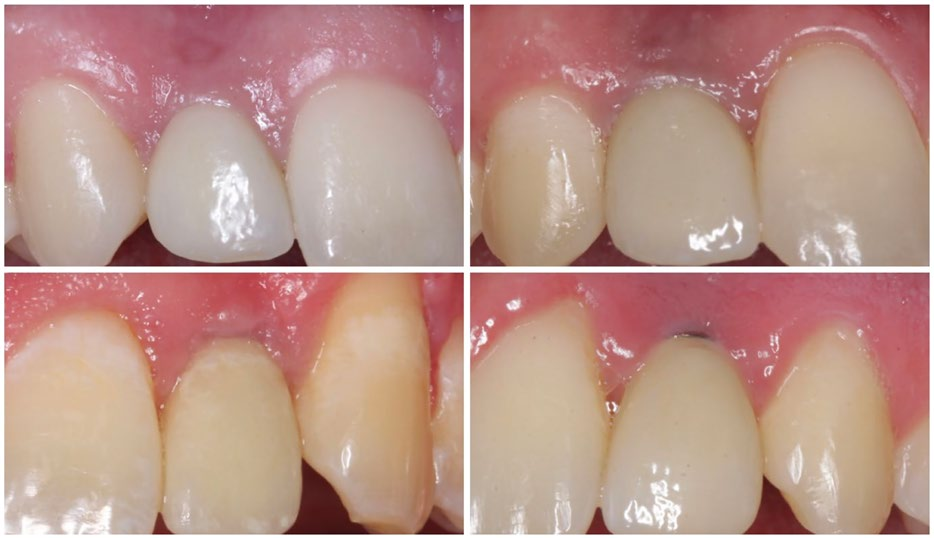
Clinical scenarios of different complications at the 5‐year follow: Presence of a mid‐buccal fistula apical to the soft‐tissue margin (a); alteration of the peri‐implant soft‐tissue color, (b), texture (c) and exposure of the Ti‐abutment in association with an apical shift of the peri‐implant soft‐tissue margin (d).

Focus of studies reporting on long‐term data on NDIs is the documentation of complications related to the reduced dimensions of the implant components (Zumstein et al. 2023): in the present study, the documented technical complications were minimal and consistent with those reported within the literature with a similar follow‐up period (Bompolaki and Bidra 2025) and did not have a significant impact on patients (Galindo‐Moreno et al. 2017). Furthermore, with respect to biological complications, no implants were diagnosed with peri‐implantitis (Renvert et al. 2018) despite the detection of a buccal fistula in three cases not associated with increased peri‐implant probing pocket depth, pus, or radiographic evidence of bone loss (Figure 4). These favorable outcomes may reflect the included young patients' low susceptibility to peri‐implant infection despite their lack of adhesion to a strict peri‐implant maintenance regime.

**FIGURE 5:**
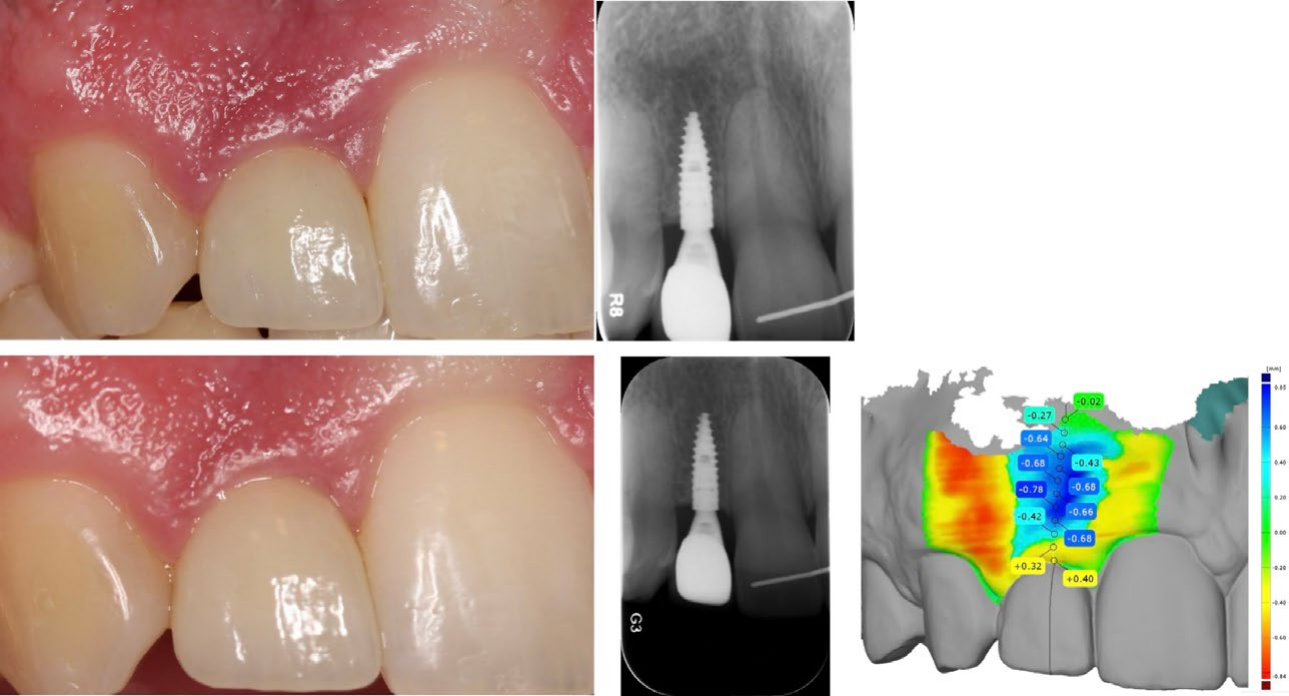
Clinical and radiographic presentation of a Ø2.9 mm implant at the 1‐ (a) and 5‐year (b) follow‐up along with the identified profilometric concave profile (c).

**FIGURE 6:**
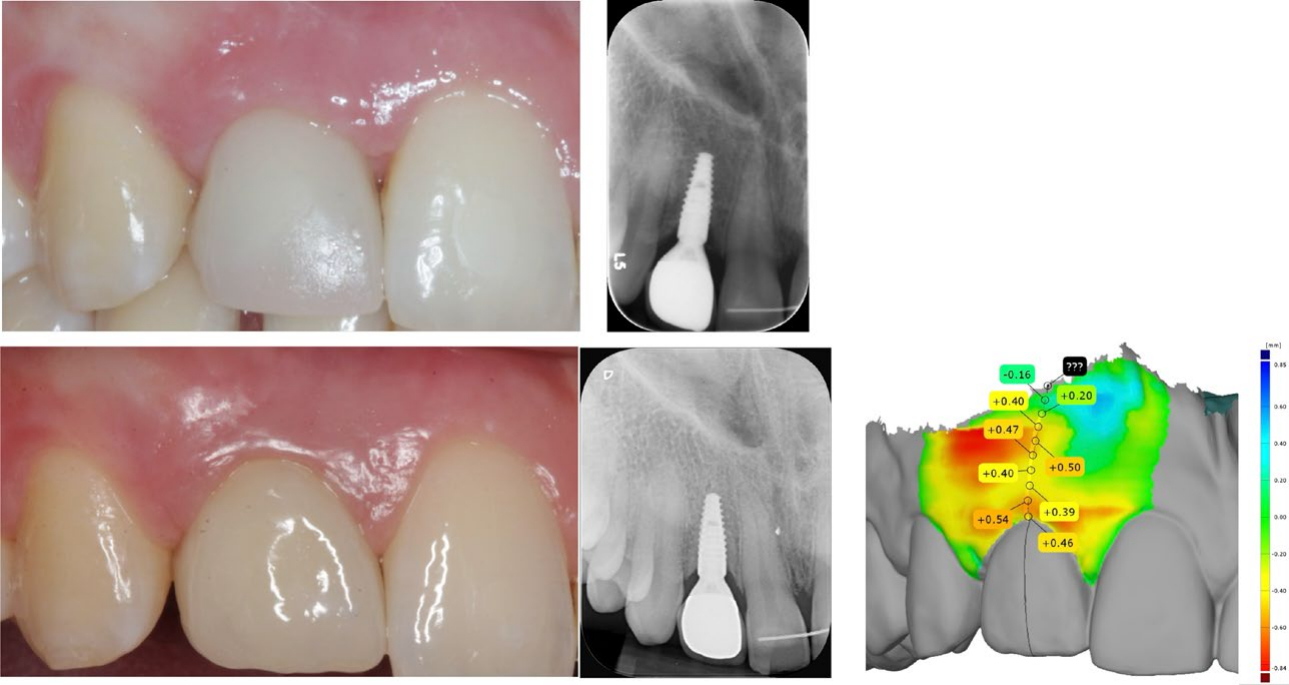
Clinical and radiographic presentation of a Ø3.3 mm implant at the 1‐ (a) and 5‐year (b) follow‐up along with the identified profilometric linear profile (c).

Esthetic outcomes, especially long‐term, are a core aspect of modern implant dentistry (Deflorian et al. 2025): the reported results support both the short as well as the mid‐term data, revealing satisfactory results in terms of symmetry, papilla fill, and level of the margin (Figures 5 and 6). However, at T4 a higher percentage of Ø3.3 mm implants (18.2%) displayed alveolar process deficiency (Score 3) compared to Ø2.9 mm implants (9.1%) as well as sub‐optimal soft‐tissue texture (Score 3: 12.1% vs. 0%) was detected, suggesting that implant diameter may play a role in preserving the alveolar process contour also in the long‐term. Finally, the detected color mismatch at T3, correlated mainly to the progressive reduction of the buccal bone wall as well as to the use of Ti‐abutment (Cosyn et al. 2021; Monje, Gonzalez‐Martin, et al. 2023; Mancini, Barootchi, et al. 2023), remained stable up to T4. One aspect which might be underlined is that these results were obtained implementing a surgical and prosthetic workflow with a classic “analogic” approach, providing direct evidence of its reliability (Baldi et al. 2020): very recently, some authors have reported, within a 2‐year prospective clinical study, similar positive results but adopting a fully digital workflow (Sorrentino et al. 2024) for the fabrication of screw‐retained zirconia single crowns on Ø3.3 mm implants, laying the foundation for a new field of research. With respect to the choice of cementation of the restorations, it should be underlined that this was done to avoid two different types of crown retention since at the time of study planning, original components to support a screw‐retained implant‐supported single crowns were not available for the Ø2.9 mm implants. Nevertheless, due to the lack of randomized clinical trials on this topic, definitive conclusions cannot be drawn.

During the last decade, the scientific interest in peri‐implant soft‐tissue evaluation in terms of esthetics, including volumetric analysis, has gained popularity (Thoma et al. 2021): classically, such analysis was performed by superimposing STL/DICOM files gathered by patients' dental casts at different time points, allowing us to quantify linear and volumetric soft‐tissue changes over time (Strauss et al. 2024). Very recently, to overcome potential distortions associated with impression materials and plaster casts, the use of STL files collected by intraoral digital scans has been proposed and validated (Mancini et al. 2024): such a technique has been implemented in the present cohort by describing three peri‐implant soft‐tissue profiles (i.e., linear, concave, and convex) and detecting a predominantly concave profiles (72.7%) within the Ø2.9 mm group, while the Ø3.3 mm group showed a more homogeneous distribution among linear (40.6%), concave (37.5%), and convex (21.9%) profiles. The interpretation of these findings might be challenging due to the lack of baseline assessment, as well as the lack of information on the soft‐tissue thickness and its potential impact on the underlying peri‐implant buccal bone resorption. Nevertheless, they represent an important reference point to monitor soft‐tissue changes in the long term (i.e., 10‐years) as well as to precisely quantify changes in the adjacent teeth position and study infra‐occlusion, which has been documented as one of the major concerns in the rehabilitation of young patients with dental implants (Klinge et al. 2021; Nilsson et al. 2019; Winitsky et al. 2021). On this aspect, it must be emphasized that infraposition, as well as changes in tooth‐implant contact points, were not systematically evaluated. Nevertheless, the analysis of the clinical pictures failed to detect significant changes up to 5 years of loading.

When focusing on patient‐reported outcomes, the presented results are confirmatory of previous long‐term studies suggesting that the significant improvements obtained at the end of the treatment remained stable up to 5 years after loading (Duong et al. 2022), despite some documented esthetic complications, providing indirect evidence of discrepancies between operators' and patients' evaluation of the esthetic outcomes (Thoma and Strauss 2022).

The present investigation accounts for some limitations which should be disclosed: first, the non‐randomized design may have introduced selection bias, as patients were allocated based on mesio‐distal space of the edentulous gap rather than through randomization. However, it should be recalled that patients' characteristics were comparable at baseline and that implant diameter has a major impact on the prosthetic emergency profile, consequently precluding the placement of a Ø2.9 mm in cases where a Ø3.3 mm would have been indicated. Secondly, the high dropout rate (i.e., 34%), although equally distributed between groups, may have affected the power analysis. In addition, it must be mentioned that the analyzed patients at 5 years were those “adherent” to the proposed follow‐up regime (i.e., check‐up visit at T2, T3 and T4); therefore, a positive interpretation bias cannot be excluded. Third, the radiographic analysis was performed on non‐standardized radiographs, providing only bidimensional information and consequently not allowing any analysis on peri‐implant buccal bone changes. Finally, the lack of systematic collection of peri‐implant bleeding on probing did not allow a precise discrimination between cases of peri‐implant health and peri‐implant mucositis.

In conclusion, both Ø2.9 and Ø3.3 mm narrow‐diameter implants for replacing congenitally missing MLIs demonstrated positive results in terms of implant survival rate, clinical, radiographic, profilometric, and patient‐reported outcomes up to 5 years. These findings support the routine clinical use of such NDIs in cases of limited MD space of the edentulous gap in the esthetic zone. However, a long‐term (10‐years) evaluation is encouraged to further document this therapeutic approach, especially considering the young age of patients frequently requiring the use of NDIs.

## Author Contributions

**Andrea Roccuzzo:** conceptualization, methodology, data curation, investigation, validation, funding acquisition, writing – original draft, writing – review and editing, supervision. **Jean‐Claude Imber:** methodology, investigation, formal analysis, writing – review and editing. **Jakob Lempert:** methodology, data curation, investigation, validation, writing – review and editing. **Leonardo Mancini:** methodology, software, formal analysis, validation, writing – review and editing. **Simon Storgård Jensen:** conceptualization, methodology, investigation, validation, supervision, funding acquisition, visualization, writing – original draft.

## Conflicts of Interest

The authors declare no conflicts of interest.

## Supporting information


**Table S1:** Profile by independent factors and covariates.
**Table S2:** clr70010‐sup‐0001‐Tables.docx. Profile by independent factors and covariates.

## Data Availability

The data that support the findings of this study are available on request from the corresponding author. The data are not publicly available due to privacy or ethical restrictions.
