# Supplementary material for: Five Year Clinical, Radiographic and Soft Tissue Profilometric Outcomes at Two Narrow‐Diameter Implants to Replace Missing Maxillary Lateral Incisors
Source: Clin Oral Implants Res. 2025 Jul 27;36(11):1412–23. doi: 10.1111/clr.70010 (PMC12598917; doi:10.1111/clr.70010)
Supplement: Supplementary file 1 — Table S1: Profile by independent factors and covariates. Table S2: clr70010‐sup‐0001‐Tables.docx. Profile by independent factors and covariates. [file CLR-36-1412-s001.docx]

Supplementary Table 1.

Profile by independent factors and covariates.

|  | **OR** | **95% CI** | **p-value** |
| --- | --- | --- | --- |
| **GROUP** |  |  |  |
| 2.9mm | 1 |  |  |
| 3.3mm | 0.23 | 0.08 – 0.62 | **0.004** |
| **WIDTH OF ALVEOLAR RIDGE** | 1.48 | 0.99 – 2.21 | 0.055 |
| **THICKNESS FACIAL BONE AFTER OSTEOTOMY** | 1.91 | 0.82 – 4.45 | 0.136 |
| **DEHISCENCE** |  |  |  |
| No | 1 |  |  |
| Yes | 0.64 | 0.07 – 5.61 | 0.684 |
| **FENESTRATION** |  |  |  |
| No | 1 |  |  |
| Yes | 0.57 | 0.20 – 1.64 | 0.300 |
| **THIN FACIAL BONE** |  |  |  |
| No | 1 |  |  |
| Yes | 0.37 | 0.14 – 0.96 | **0.041** |
| **AUTOGENOUS BONE GRAFT** |  |  |  |
| No | 1 |  |  |
| Yes | 0.42 | 0.16 – 1.11 | 0.080 |
| **USE BONE SUBSTITUTE** |  |  |  |
| No | 1 |  |  |
| Yes | 0.36 | 0.13 – 0.96 | **0.042** |

Results of simple ordinal logistic regression (OR and 95%CI, p-value)

Supplementary Table 2.

Profile by independent factors and covariates.

|  | **OR** | **95% CI** | **p-value** |
| --- | --- | --- | --- |
| **GROUP** |  |  |  |
| 2.9mm | 1 |  |  |
| 3.3mm | 0.25 | 0.08 – 0.76 | **0.015** |
| **WIDTH OF ALVEOLAR RIDGE** | 1.48 | 0.94 – 2.32 | 0.087 |
| **THIN FACIAL BONE** |  |  |  |
| No | 1 |  |  |
| Yes | 0.58 | 0.08 – 4.41 | 0.603 |
| **AUTOGENOUS BONE GRAFT** |  |  |  |
| No | 1 |  |  |
| Yes | 1.02 | 0.25 – 4.15 | 0.977 |
| **USE BONE SUBSTITUTE** |  |  |  |
| No | 1 |  |  |
| Yes | 0.86 | 0.08 – 9.63 | 0.904 |

Results of multiple ordinal logistic regression (OR and 95%CI, p-value)
